# Psychomotor Delay, a Possible Rare Presentation of Moyamoya Disease

**Published:** 2011-09-25

**Authors:** M. R. Ashrafi, H. Alizadeh, S H. Yazdani, M. Mohseni, M. Mohamadi

**Affiliations:** 1 Associate Professor, Department of Pediatric Neurology, Children’s Medical Center, Tehran University of Medical Sciences, Tehran, Iran; 2 Assistant Professor, Department of Pediatric Radiology, Children’s Medical Center, Tehran University of Medical Sciences, Tehran, Iran; 3 Resident of Cardiology, Department of Cardiology, Modarres Hospital, Shahid Beheshti University of Medical Sciences, Tehran, Iran; 4 Resident of Neurosurgery, Department of Neurosurgery, Imam Khomeini Hospital, Tehran University of Medical Sciences, Tehran, Iran; 5 Professor, Department of Pediatric Neurology, Children’s Medical Center, Tehran University of Medical Sciences, Tehran, Iran

**Keywords:** Moyamoya, Child, Developmental Delay, Cerebrovascular Disorders, Magnetic Resonance Angiography

## Abstract

Moyamoya disease is a rare, chronic cerebrovascular occlusive disease of unknown etiology. It is characterized by progressive stenosis of the arteries of the circle of Willis leading to ischemic strokes in young people and cerebral hemorrhage, which is more frequent in adults. Secondarily, an abnormal network of fine collateral vessels arises at the base of the brain. The term moyamoya refers to the angiographic appearance of the cerebral vasculature. We present such a disease in an 18-month-old Iranian girl with global developmental delay, which is a very rare presentation of moyamoya disease. She was diagnosed by magnetic resonance imaging (MRI) and magnetic resonance angiography (MRA).

## Introduction

Moyamoya disease was initially described as a disease in Japan, with approximately a total number of 3900 cases in 1995 and an annual incidence of 0.35 per 100000 in this population [1]. Japanese and Koreans have higher incidence than Caucasians.

It is more common in the first decade of life with a male to female ratio of 1:1.7 [2][3]. Moyamoya disease has an unknown etiology despite extensive studies over a long period [4].

Moyamoya disease is a chronic occlusive cerebrovascular disorder characterized by progressive stenosis of the arteries of the circle of Willis first reported by Japanese surgeons Takeuchi and Shimizu as hypoplasia of the bilateral internal carotid arteries in 1957 [5]. The process initially involves the intracranial internal carotid artery and progresses to the involvement of the middle, anterior and posterior cerebral arteries and a coexisting abnormal vascular network at the base of the brain. The disease was first named as moyamoya in 1969 by Suzuki and Takaku [6].

Collateral vessels at the base of the brain make a so called “puff of smoke” appearance which is the angiographic hallmark of the disease [4]. In histopathology, stenosis or occlusion of the main brain arteries are noted with fibrocellular thickening of the intima containing proliferated smooth muscle cells and tortuous often duplicated internal elastic lamina. The atheromatous plaques in the arterial wall are unusual [2].

The incidence of moyamoya disease is high among Japanese and Koreans and far lower in other ethnic groups. In children, the usual clinical manifestations are ischemic events, hemiparesis being the most common clinical manifestation. As an initial manifestation, children with moyamoya disease frequently develop seizures or focal motor signs because of cerebral ischemia. [7][8][9][10]. Children may have hemiparesis, monoparesis, sensory impairment, involuntary movements, headaches, dizziness, or seizures. Mental retardation or persistent neurologic deficits may be present.

We report psychomotor developmental delay, a very rare possible presentation of moyamoya disease.

## Case Presentation

Here we present an 18-month-old girl with psychomotor delay. She was referred at the age of 3.5 months due to poor head control and retrocollis. She was the first child of consanguineous parents with an unremarkable family history. Her mother underwent cesarean section due to fetal distress and pre-eclampsia at 38 weeks of gestational age, but birth history was unremarkable with a normal Apgar score. Her birth weight and head circumference (HC) were 2450 gm and 32 cm, respectively. She was discharged after two days and only mild jaundice was seen that needed no intervention. Our first visit revealed head lag on traction maneuver and according to the parents report she had no social smile. Her head circumference was 40 cm and no dysmorphic features were found. Global developmental delay was suggested and the workups were carried out.

**Fig. 1:**
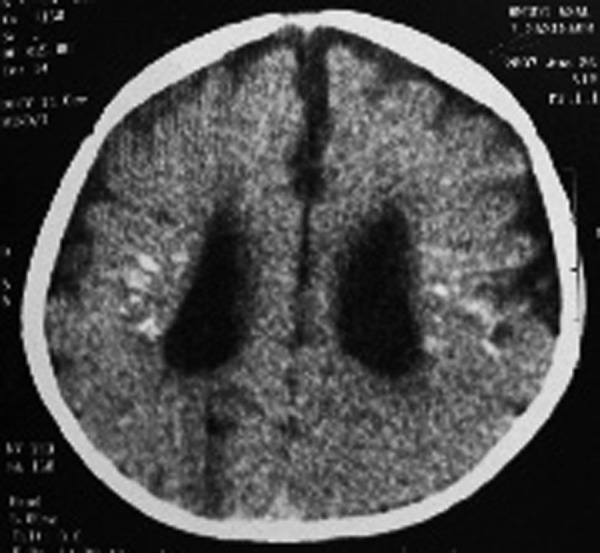
An 18-month-old girl with psychomotor delay. Brain CT scan without contrast reveals mild ventriculomegaly with symmetric punctate and linear periventricular calcifications

Brain computerized tomography (CT scan) without contrast injection was performed. It showed mild ventriculomegaly, symmetric punctate and linear periventricular calcifications, hyperdense foci in both thalami and subtle linear calcification around the right central sulcus. No obvious structural abnormality was seen at that time (Fig. 1). Auditory brainstem response and electroencephalography (EEG) were normal.

Arterial blood gas study (pH=7.39, HCO3=22.4), urine amino acid and carbohydrate chromatography and also plasma amino acid chromatography (HPLC method) had normal results.

Blood ammonia, pyruvate and lactate levels are shown in Table 1 and TORCH study results were as demonstrated in Table 2.

According to the increased blood lactate, empiric treatment with vitamins (B1, B6 and biotin) and carnitine was started with the impression of mitochondrial disorder. At the age of 6 months, head lag, truncal hypotonia and fisting of the hands were present. HC was 42 cm. Her blood lactate level reduced to 14.8 mg/dl. At this time, she underwent physiotherapy. At the age of 9 months, head lag was still present, HC was 43 cm but eye fixation and following were seen. At 13 months of age HC was 44 cm. Spasticity of lower extremities, brisk deep tendon reflexes, ankle clonus and fisting of the hands were detected. No babbling was done by the infant. Parents did not report any seizure activities and the second EEG was normal.

**Table 1:** Blood Ammonia, Pyruvate and Lactate Levels

Blood Ammonia	36.8 umol/L	Normal Value: 10-47
Pyruvate	0.8 mg/dl	Normal Value: 0.3-0.9
Lactate	42.9 mg/dl	Normal Venous Value: 5-20

**Table 2:** TORCH Study Results

Anti Toxoplasma Gondii IgM	Negative
Anti Toxoplasma Gondii IgG	4.0 IU/ml (Negative< 6.0)
Rubella IgM Elisa:	Negative
Rubella IgG Elisa	7.0 IU/ml (Negative<9)
CMV IgM Elisa	Negative
CMV IgG Elisa	2.8 IU/ml (Positive>1.1)
HSV 1 (IgG)	0.4 IU/ml (Negative<0.9)
HSV1, 2 (IgM)	Negative

**Fig. 2:**
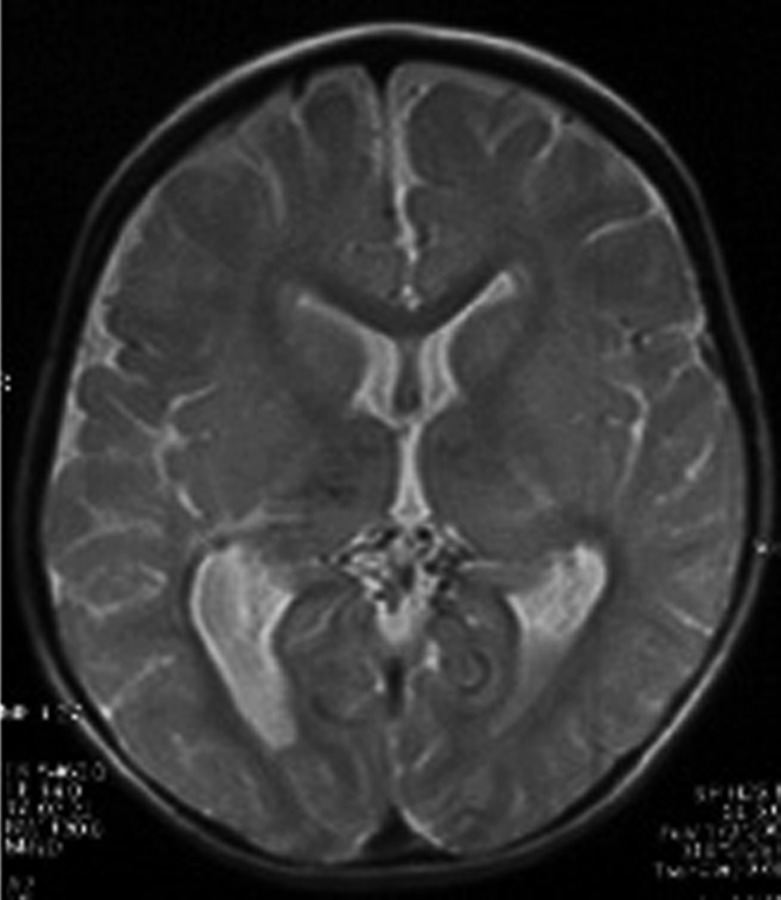
Axial proton density spin echo MRI shows abnormal tiny tubular structures with signal void suspected as small collaterals in the supracerebellar and retropulvinar cisterns associated with similar findings in both thalami

Brain MRI without paramagnetic contrast revealed bilateral symmetric periventricular and parietal abnormal signals, T1 and T2 prolongation with dilatation of lateral ventricles in the trigones suggesting periventricular leukomalacia (PVL) and secondary gliotic changes. Proton density axial planes showed several signal void and small tubular structures in the supracerebellar cistern and around the thalami associated with bilateral hypoplastic internal carotid arteries (ICAs) that suggested tiny collateral vessels (Fig. 2).

**Fig. 3:**
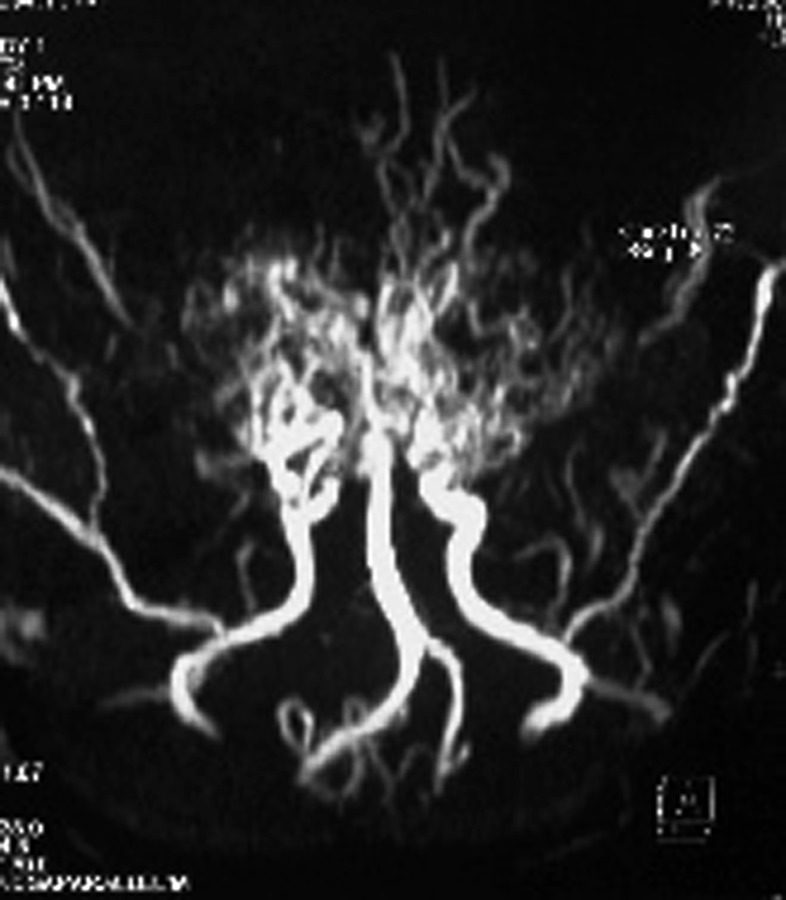
Prominent thalamoperforating collaterals (puff of smoke), absence of supraclinoid portions of both ICAs and bilateral hypertrophied leptomeningeal vessels, especially middle meningeal arteries are noted in the MRA study.

Based on these findings, a magnetic resonance angiography (MRA), time-of-flight technique (TOF), was requested. This technique revealed prominent thalamoperforating collaterals (puff of smoke), absence of supraclinoid portions of both ICAs and bilateral hypertrophied leptomeningeal vessels especially the middle meningeal arteries (Fig. 3), which confirmed the diagnosis of moyamoya disease.

Echocardiography, G banding karyotyping and sickle prep were normal. During her last visit at the age of 18 months, HC was 45 cm and she still had head lag. Palmar grasp was absent, but babbling and social smile were seen.

## Discussion

Moyamoya disease is a chronic occlusive cerebrovascular disorder first reported by Japanese surgeons. It is characterized by progressive stenosis of the arteries of the circle of Willis.

Due to the higher incidence of this disease in orientals, the low incidence in Caucasians and the presence of familial cases (7-10% of cases) multifactorial inheritance is possible [2][3]. In some studies, the risk of the disease was estimated about 40 times greater in children with a first-degree relative affected by the disease [3]. Although the great majority of the patients have primary disease and do not have recognized risk factors, some cases have secondary forms (moyamoya syndrome).

In children, the usual clinical manifestations are due to ischemic events with hemiparesis being the most common clinical manifestation. As an initial manifestation, children with moyamoya disease frequently develop seizures or focal motor signs because of cerebral ischemia. “Children may have hemiparesis, monoparesis, sensory impairment, involuntary movements, headaches, dizziness or seizures. Mental retardation or persistent neurologic deficits may be present. On the other hand, in adult patients, frequent initial symptoms are headache, sudden loss of consciousness or focal motor signs. In adults, cerebral hemorrhage may occur. [7][8][9][10]

Our reported case is an 18-month-old girl that came into observation due to psychomotor delay. She had no focal neurologic deficits or seizure disorders. Delay is said to exist when a child does not reach developmental milestones at the expected age considering the adequate margin for the broad variation among normal children. [11] Global developmental delay can be operationally defined as a significant delay in two or more developmental domains (gross motor, fine motor, cognition, speech/language and personal/social) [12]. Global developmental delay is not a common presentation for moyamoya and is not reported in the literature as the sole presentation for moyamoya. Motor (gross, fine) and speech delay were seen in our case that suggest global developmental delay.

Clinical diagnosis depends on cerebral angiography. The diagnostic criteria are stenosis or occlusion of the terminal portions of the internal carotid artery and proximal portions of the anterior or middle cerebral arteries with the characteristic “puff of smoke” appearance.

Due to the risks of invasive procedures and radiation, MRA is now the preferred method of investigation of moyamoya disease [13].

Our reported case was referred due to developmental delay and therefore, underwent workup. Brain CT showed atrophy and calcifications suggesting prenatal or perinatal insults. TORCH studies were unremarkable. Although pre-eclampsia and fetal distress were reported, the patient was delivered via cesarean section with no evidence of asphyxia. She had no family history of a similar disease or recognizable predisposing factors for moyamoya syndrome. Increased blood lactate was the only abnormal laboratory finding in our case. Although increased blood lactate may be considered as a cause of brain atrophy and calcification, it cannot explain the moyamoya appearance of cerebral vasculature. The hypothesis that brain calcification and atrophy may be due to intrauterine hypoxia and moyamoya disease or hyperlactatemia may be a predisposing factor for moyamoya syndrome need to be studied.

The natural course of moyamoya disease is progressive and the affected children are at increased risk of intracranial hemorrhage with an associated high mortality. Early diagnosis and treatment of moyamoya disease in children is essential to minimize residual mental and physiologic deficits. Medical treatment is targeted toward elimination of clotting and it usually includes acetylsalicylic acid, calcium channel blockers, vasodilators, steroids, anticoagulants and antibiotics, all of which remain ineffective. [14]

This fact would support the need for surgical intervention for revascularization of the ischemic brain by creating collateral pathways [2][7]. Surgical revascularization of the brain is the preferred treatment for moyamoya disease in children including superficial temporal to middle cerebral artery anastomosis, encephaloduroarteriosynangiosis and encephalomyosynangiosis.[15] Our case was a candidate for surgical intervention and antiplatelets were administered.

Moyamoya disease is rare in the Iranian population and its presentation as developmental delay is rare in the world.
